# Living with voracious roommates: Factors that explain isotopic niche variation in a mixed colony of insectivorous bats

**DOI:** 10.1002/ece3.10939

**Published:** 2024-03-17

**Authors:** Isaac Peña‐Villalobos, Catalina B. Muñoz‐Pacheco, Martín A. H. Escobar, Fabian M. Jaksic, Pablo Sabat

**Affiliations:** ^1^ Departamento de Ciencias Ecológicas, Facultad de Ciencias Universidad de Chile Santiago Chile; ^2^ Laboratorio de Células troncales y Biología del Desarrollo, Departamento de Biología, Facultad de Ciencias Universidad de Chile Santiago Chile; ^3^ Grupo de Ecología, Naturaleza y Sociedad (GENS), Departamento de Gestión Forestal y su Medio Ambiente, Facultad de Ciencias Forestales y de la Conservación de la Naturaleza Universidad de Chile Santiago Chile; ^4^ Escuela de Arquitectura del Paisaje Universidad Central de Chile Santiago Chile; ^5^ Facultad de Ciencias de la Naturaleza Universidad San Sebastián Santiago Chile; ^6^ Center of Applied Ecology and Sustainability (CAPES) Santiago Chile; ^7^ Millennium Nucleus of Patagonian Limit of Life (LiLi) Valdivia Chile

**Keywords:** isotopic ecology, moonlight, *Myotis chiloensis*, niche segregation, *Tadarida brasiliensis*

## Abstract

Theory predicts that in resource‐limited environments, coexisting species may overlap their niche dimensions but must differ in at least one to avoid competitive exclusion. Specifically, it has been suggested that the coexistence of competing species within a guild, could be sustained with mechanisms of resource partitioning, such as segregation along a trophic dimension. Among the most gregarious mammals are bats, which present diversification in their diet based on habitat choice and body size. Despite differences that could explain specialization in prey selection, there are insufficient studies that explore food overlap in mixed bat colonies and the factors that determine the selection of prey, both at intra‐ and inter‐specific levels. To fill this gap, we analyzed the isotope signal (δ^13^C and δ^15^N) in feces collected in a mixed colony of *Tadarida brasiliensis* and *Myotis chiloensis*. To understand how several factors could influence these isotopic signals, intrinsic explanatory variables were analyzed, including body mass, body length, age, and sex. Also, extrinsic variables were analyzed, including monthly temporality and moonlight intensity. Our findings support age‐dependent specialization in *M. chiloensis*, with a significant role of moonlight intensity and sex on δ^15^N. In *T. brasiliensis*, we identified a significant effect of size, sex, and ear length on δ^15^N. Our analysis indicates that both species of bats experience diverse degrees of overlap through austral summer months, affected by several factors that explain the variability in their fecal isotopic signals.

## Graphical abstract

This study analyzed the isotope signal in feces collected from a mixed colony of *Tadarida brasiliensis* and *Myotis chiloensis* to explore feeding overlap and the factors that determine prey selection. The results showed that age‐dependent specialization exists in *Myotis chiloensis*, with a significant role in moonlight intensity and sex. The study also identified an important effect of size, sex, and ear length in *Tadarida brasiliensis*. Both species of bats experience diverse degrees of overlapping through the months of austral summer, affected by several factors that explain the variability in their feces isotopic signals.

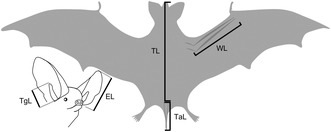

## 1 INTRODUCTION

Quantifying ecological niches and their overlap between species has regained the interest of ecologists (e.g., Bearhop et al., 2004; Bobadilla et al., 2022; Costa‐Pereira et al., 2018; Schirmer et al., 2020). Niche theory (Hutchinson, 1957) predicts that when resources are limited, coexisting species may overlap their niches on several of their axes but must differ on at least one to avoid competitive exclusion. For instance, the coexistence of potentially competing species within a guild could be sustained with mechanisms of resource partitioning such as the segregation on some trophic dimension (Arlettaz et al., 1997; Ruadreo et al., 2018). Understanding the degree of specialization or generalization in individuals and species is important because it can inform problems as diverse as the evolution of resource use in changing environments (Bolnick et al., 2003; Emslie & Patterson, 2007), the tolerance of a population to changing resource levels, its ability to resist competition, and its response to other factors that may affect population size (Wittwer et al., 2015).

Chiropterans are the most gregarious mammals, forming colonies of up to thousands of individuals, even in communities with multiple species (Afonso et al., 2017). Previous studies on bats have generally focused on the morphological differences that might explain the partitioning of trophic resources and the competition between species of microchiropterans (Aldridge & Rautenbach, 1987; Arlettaz, 1999; Arlettaz et al., 1997), identifying a strong component of habitat choice and body size on the type of prey consumed. Among the traits that could be determinant in the foraging capacity of bats—and therefore in their niche segregation—ecomorphological approaches have attempted to explain differences in food selection or processing (Swartz et al., 2003). It has been concluded that the wings, ears, brain, jaws, and teeth of bats are indicative of their ecological functioning (Findley & Wilson, 1982). Among these features, wing morphology and wing loading (i.e., the ratio between the body mass of the animal and the surface of the wing) have been proposed as adequate predictors of flight height and maneuverability, which in turn have been associated with variability of energetic and ecological characteristics (Canals et al., 2001).

In Chile, the mouse‐tailed bat *Tadarida brasiliensis* and the mouse‐eared bat *Myotis chiloensis* are insectivorous species that feed mainly on arthropods and usually form mixed colonies (Ossa & Rodríguez‐San Pedro, 2015). Canals et al. (2001) reported that *M. chiloensis* has a low wing load, which enables high maneuverability in flight, displayed during foraging periods of 3 h daily. In contrast, *T. brasiliensis* has a high wing load, which translates to high‐speed flights but with low maneuverability. Besides these morphological differences, several studies have documented distinct selectivity over different species of insects, such as mosquitoes for *M. chiloensis* and moths for *T. brasiliensis* (Galaz & Yáñez, 2006; Koopman, 1967; Mann, 1978; Olmedo et al., 2021; Silva & Fleck, 1976).

Despite previous cases searching for differences that could explain specialization in prey selection by bats, there are still few studies that explore the feeding overlap in mixed bat colonies, and even fewer that assess the possible factors underlying prey selection, both at intra‐ and inter‐specific levels (e.g., Arlettaz et al., 1997; Ashrafi et al., 2011; Siemers & Swift, 2006). To fill this gap, we analyzed the isotopic niche (Newsome et al., 2007) as a proxy of trophic niche along with intrinsic and extrinsic factors associated with the dietary preferences in a mixed colony of *T. brasiliensis* and *M. chiloensis* in Chile. For characterizing the trophic preferences of these bats, we used stable carbon and nitrogen isotopic signatures found in feces, given their wide applicability and usefulness in understanding dietary habits across various animal species, including chiropterans (Painter et al., 2009). Stable isotope ratios offer valuable insights into an animal's dietary ecology and trophic interactions because they are closely linked to dietary sources. For instance, the ratio of heavy to light stable nitrogen isotopes (^15^N/^14^N) progressively increases with trophic level (Fry, 1988; Minagawa & Wada, 1984; Wada et al., 1987) reflecting a bionomic axis (Newsome et al., 2007). Stable carbon isotopes (^13^C/^12^C) chiefly reveal the primary carbon sources within a food web (e.g., C3 vs. C4 plants; Rounick & Winterbourn, 1986). Comparing carbon δ^13^C and nitrogen δ^15^N values among consumers (i.e., the isotopic niche, Newsome et al., 2007) provides a quantitative indication of an organism's trophic niche, allowing a comprehensive exploration of this niche in bat species.

Studies in other taxa have reported that the overlap in isotopic niches and niche space expansion correlates with dietary diversity (Krumsick & Fisher, 2019; Stewart et al., 2021). This suggests that dietary diversification leads to distinct isotopic niches among species or individuals, while a lack of dietary diversification can result in high isotopic niche overlap and reflect potential competition for the same resources. In this vein, indications exist of dietary specialization in bats, possibly influenced by factors such as wing morphology or sex (Arango‐Diago et al., 2020; Magalhães de Oliveira et al., 2020). Additionally, environmental factors, including moonlight and seasonality, have the capacity to alter foraging patterns of bats (Appel et al., 2017; Fenton et al., 1977; Klingbeil & Willig, 2010; Saldaña‐Vázquez & Munguía‐Rosas, 2013; Vásquez et al., 2020).

To gain a better understanding of the trophic use and isotopic niche width in a bat community, we conducted an analysis of intrinsic explanatory traits, including body mass, body length, age, and sex. Additionally, we examined monthly temporality and moonlight intensity as extrinsic variables, which in turn are dependent on environmental and temporal settings. We hypothesized that given the mixed colonies formed by *T. brasiliensis* and *M. chiloensis*, there should be dietary diversification driven by morphological differences, to avoid potential competitive exclusion.

## 2 METHODS

### 2.1 Sampling and measurement

*T. brasiliensis* (Vespertilionidae) is a medium‐sized bat (head and body length 9–12 cm; Galaz et al., 2020) distributed from Canada to Chile (Eger, 2008; Simmons, 2005). It is an insectivorous species, which feeds mainly on Coleoptera, Hemiptera, Homoptera, Hymenoptera, Lepidoptera, and Neuroptera (Galaz & Yáñez, 2006; Silva & Fleck, 1976). In contrast, *M. chiloensis* (Molossidae) is a small‐sized species (head and body length 7–10 cm; Galaz et al., 2020) endemic to the southern cone of South America, which occurs only in Chile and Argentina (Simmons, 2005; Wilson, 2008), feeding mainly on insects, chiefly Diptera (Mann, 1978).

We collected fecal samples from a mixed colony of *T. brasiliensis* and *M. chiloensis* in the Quilapilún Ethnobotanical Park (33°05′30.62″ S, 70°43′52.82″ W). The study area is dominated by sclerophyllous scrub and forest in the Metropolitan Region of Chile, a plant formation adapted to Mediterranean‐type climatic conditions. Within a 1 km buffer from the capture zone as the center, 10% of the surface corresponds to densely covered forest, 13% to agricultural cultivar, and 77% to thorny scrubland. This mixed habitat type is suitable for many species of bats, including those that prefer open spaces or forested areas. The climate is characterized by cool and rainy winters and warm and dry summers (Gajardo, 1994).

We performed captures in an abandoned house during the austral summer season (from January 12 to March 4, 2019), with six campaigns lasting 1–2 days each and a 10‐day interval between campaigns. We used four mist nets (two measuring 10 × 2.5 m, one measuring 6 × 2.5 m, and one measuring 3 × 2.5 m), along with one harp trap (4 × 2 m) placed near doors and windows. These traps were active from 20:00 to 03:00 h, with inspections conducted every 20 min (Puelma‐Diez et al., 2021).

**FIGURE 1:**
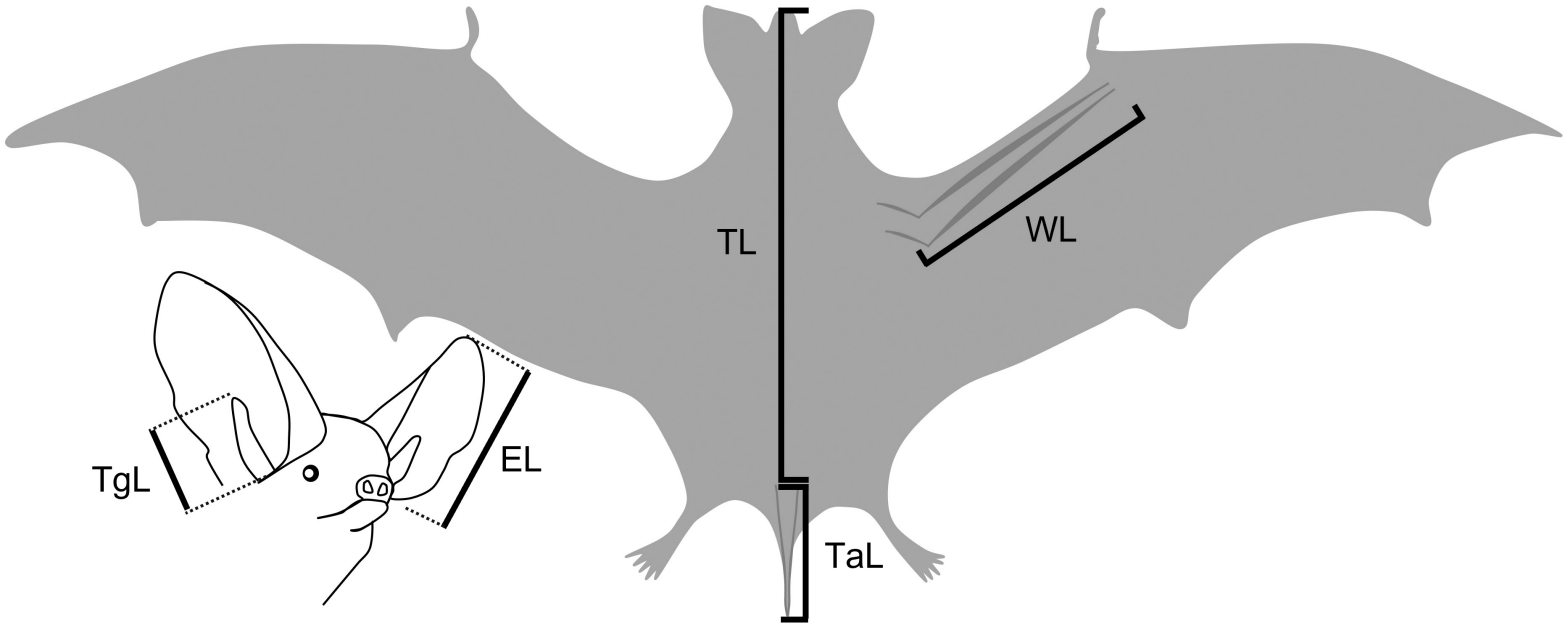
Morphometric variables measured in two sympatric bat species (*Myotis. chiloensis* and *Tadarida brasiliensis*) in Chile. Total length (TL), wing length (WL), tail length (TaL), tragus length (TgL), ear length (EL).

Once captured, each specimen was marked, measured, weighed, sexed, and its feces were collected and weighed. Immediately after, fecal samples were dried at room temperature and kept for 12 months at 25°C in centrifugation tubes (1.5 mL) for later analysis. Total length (TL) was measured using a ruler, while left‐wing length (WL), tail length (TaL), tragus length (TgL), and left ear length (EL) were measured with a caliper (±0.01 mm; see Figure 1). We marked individuals with MiniHPT8 subdermal chips (Biomark, Inc.) in the lower lumbodorsal region for identification (Escobar et al., 2022). During the collection period, a total of 262 individuals was captured, with 176 belonging to the species *M. chiloensis* and 86 to *T. brasiliensis*. Due to budget and time constraints, a subsample of 71 individuals was chosen for analysis, consisting of 46 *M. chiloensis* (35 females: 25 adults, 7 juveniles, 3 fledglings; 11 males: 1 adult, 8 juveniles, 2 fledglings) and 25 *T. brasiliensis* (11 females: 9 adults, 2 juveniles; 14 males: 12 adults, 2 juveniles). Species identification followed Rodríguez‐San Pedro et al. (2014), and we determined age (fledgling, juvenile, or adult) based on wing bone epiphyseal ossification (De Paz, 1986; Monadjem et al., 2018). People involved in capture and handling were immunized against rabies.

Data for moonlight intensity were obtained from the internet (www.timeanddate.com) for each day of capture, based on the percentage of sunlight incident on the moon that was reflected.

### 2.2 Isotope analysis

Feces were cleaned in distilled water for 24 h to remove urine remains. They were then dried in a laboratory oven at 70°C for a week, ground up manually, and stored in Whatman filter paper. Because of our lack of knowledge of the fecal lipid content, samples were subjected to lipid extraction. This decision was based on the observation that lipids tend to be more depleted in ^13^C compared to tissues. By doing that, we mitigated the potential introduction of biases in our findings (Salvarina et al., 2013). Samples were defatted for 2 h using a Soxhlet apparatus and petroleum ether and dried for 3 days at 60°C. After that, 0.5–0.6 mg of dried feces was weighed in tin capsules, and carbon (δ^13^C) and nitrogen (δ^15^N) isotope values were measured on a Costech 4010 elemental analyzer coupled to a Thermo Scientific Delta V Plus isotope ratio mass spectrometer. Isotope measurements were conducted at the Stable Isotope Ecology Laboratory & UA Stable Isotope Facility, Universidad de Antofagasta (Chile), and values were reported using standard delta (δ) notation in parts per thousand (‰) as δX = (*R*_sample_/*R*_standard_ − 1), where *R*_sample_ and *R*_standard_ are the ratios of the heavy to light isotope of the sample (e.g., ^13^C/^12^C) and the reference, respectively. The internationally accepted references are Vienna Pewee Belemnite for δ^13^C and atmospheric N_2_ (AIR) for δ^15^N. Within‐run precision (SD) for both δ^13^C and δ^15^N was estimated via analysis of internal reference materials and found to be ≤0.2‰ for both isotopes.

### 2.3 Statistical analysis

To identify potential associations between isotopic signatures of each individual with regard to intrinsic (body measurements, body mass, species, age, and sex) and extrinsic factors (monthly temporality and moonlight intensity), we evaluated the relationship of these variables by means of Pearson's correlation coefficient and a permutation test. Additionally, we compared the body measurements between the two species using one‐way and factorial ANOVA and ANCOVA (using body weight as covariates). All analyses considered an alpha value of 0.05. To explore the influence of intrinsic and extrinsic factors on δ^13^C and δ^15^N, we utilized generalized linear mixed models (GLMMs) with a normal distribution. Our approach involved examining all potential linear combinations involving up to three variables. To address variability, we included sex, month, species, and sampling date as random effects. Additionally, we conducted separate analyses for each species and pooled the data. These were performed to address the variability and correlation within the data arising from the grouping or clustering of observations. Following that, we identified the two models that demonstrated the best fit to our data through the delta AICc (corrected Akaike's Information Criterion), utilizing the AICcmodavg package for R (Mazerolle, 2023). This criterion measures the difference in AICc score between the best model and the model under comparison.

We estimated the isotopic niche width of each species using standard ellipse areas corrected for small sample sizes (SEA_C_; Jackson et al., 2011). To compare isotopic niche widths across species and seasons, we constructed Bayesian standard ellipse areas (SEA_B_, Jackson et al., 2011). This method—using Markov chain Monte Carlo simulations with 10,000 iterations for SEA_B_—allowed us to calculate the proportion of posterior draws that are smaller between two groups, which was used as the probability (*p*) that one group has a smaller isotopic niche width than the other (Jackson et al., 2011). SEA_C_, SEA_B_, and overlap between SEA_C_ were estimated using Stable Isotope Bayesian Ellipses in R (SIBER; Jackson et al., 2011). For graphical representations, we used SEA_C_.

## 3 RESULTS

### 3.1 Morphometric comparisons

As expected from previous studies (e.g., Canals et al., 2001; Schirmer et al., 2020), morphological dimensions relative to body mass were different between the two species. Total length and wing length relative to body mass were larger in *T. brasiliensis*, whereas ear and tail lengths did not exhibit significant differences between the species (Table 1). No effect of the interaction species × sex was found for wing length (*F*_(1,59)_ = 0.36, *p* = .55), total length (*F*_(1,59)_ = 0.01, *p* = .94), tail length (*F*_(1,58)_ = 0.54, *p* = .46), ear length (*F*_(1,57)_ = 0.04, *p* = .84), or body mass (*F*_(1,64)_ = 0.76, *p* = .39). After correlating capture date and body mass, a significant effect was found for *M. chiloensis*, which were heavier from January to March for both males (*r* = .967; *p* < .001) and females (*r* = .843; *p* < .001). In contrast, only females of *T. brasiliensis* exhibited a negative association between body mass and capture date (females: *r* = −.633; *p* = .037; males: *r* = −.359; *p* = .207). Additionally, in *M. chiloensis*, we observed a positive correlation between EL and TgL (*r* = .464; *p* = .004) and a marginally significant one between EL and WL (*r* = .313; *p* = .059). In *T. brasiliensis*, ear length (EL) was not associated with any other morphological feature, neither in pooled data nor by sex. The correlations for body mass in pooled data were *r* = .076, *p* = .717; for body mass in males: *r* = .061, *p* = .835; and for body mass in females: *r* = .006, *p* = .985. Similarly, the correlations for wing length (WL) in pooled data were *r* = .027, *p* = .899; for WL in males: *r* = .217, *p* = .457; and for WL in females: *r* = −.132, *p* = .700.

**TABLE 1:** Morphometric comparison between pooled data of *Tadarida brasiliensis* and *Myotis chiloensis*, from a mixed colony in Chile.

Morphological variables	*Tadarida brasiliensis*		*Myotis chiloensis*		Statistical comparison	
	*n*	Mean ± SD	*n*	Mean ± SD	*F*_(df)_	*p*‐value
WL (mm)	25	43.64 ± 1.27	39	37.75 ± 1.931	19.30_(1,61)_	<.001
TL (mm)	25	63.72 ± 3.97	39	51.44 ± 4.32	7.15_(1,61)_	.01
TaL (mm)	25	101 ± 0.00	39	99 ± 12.49	1.16_(1,60)_	.29
TgL (mm)	n/d		38	5.45 ± 0.85	n/a	
EL (mm)	25	11.42 ± 1.17	37	10.89 ± 1.44	0.001_(1,59)_	.97
Body mass (g)	25	13.21 ± 1.4	43	6.34 ± 1.4	382.13_(1,66)_	<.001

*Note*: ANCOVA results are presented for length and ANOVA for body mass.

Abbreviations: Total length (TL), wing length (WL), tail length (TaL), tragus length (TgL), ear length (EL). No data (n/d). Not applicable (n/a).

### 3.2 Isotopic niches

**FIGURE 2:**
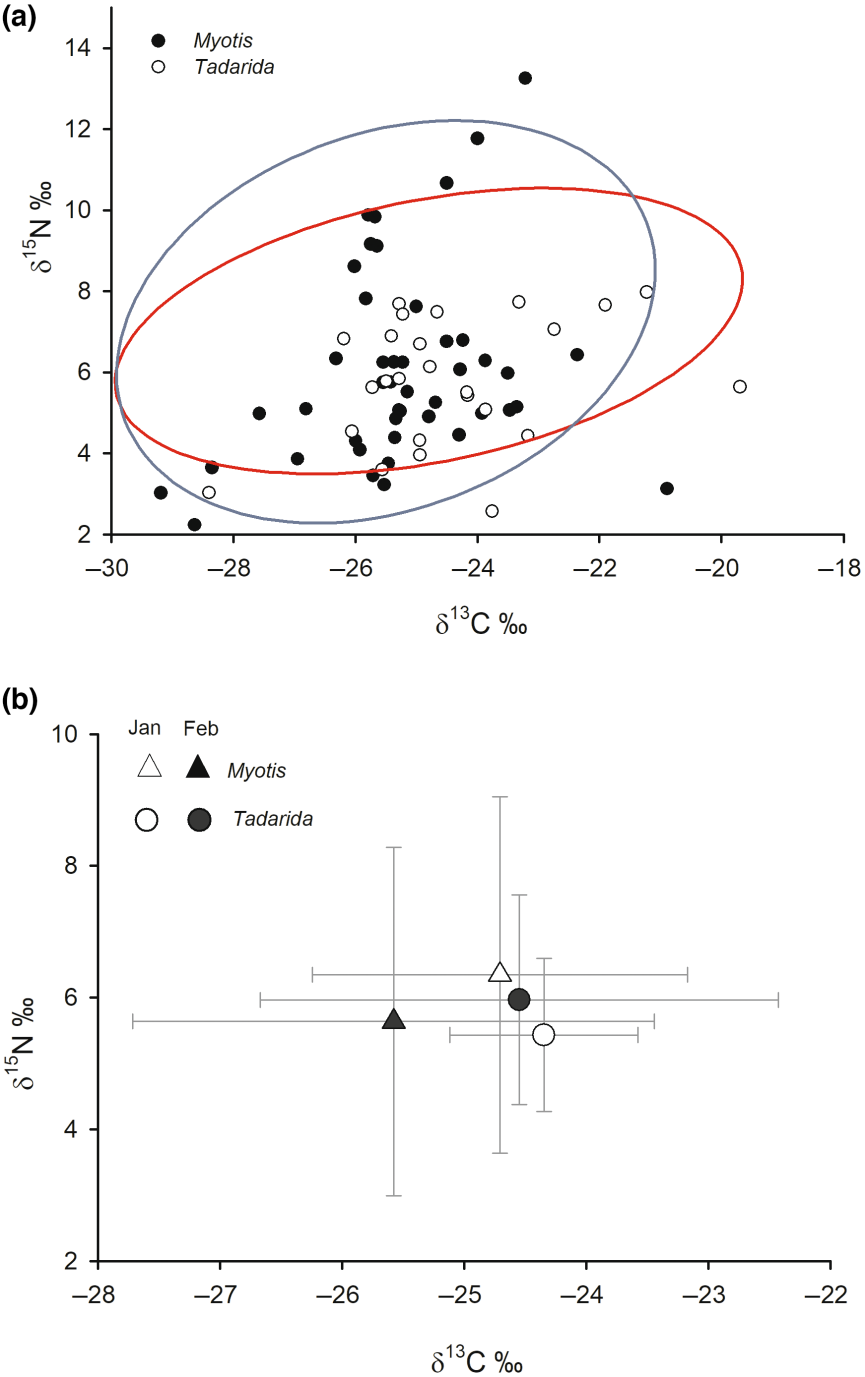
(a) Standard ellipses corrected for small sample size (SEA_C_) representing two sympatric bat species (*Myotis chiloensis* and *Tadarida brasiliensis*) in Chile. (b) Stable isotope ratios of δ^15^N and δ^13^C (mean ± SD) from feces over two consecutive months during the austral summer.

When the standard ellipse areas were constructed using the full data set, we found an overlap of 87% between the isotopic niche (SEA_C_) of the two species. Further, we found no significant differences (*p* = .396) in the size (i.e., the mode) of SEA_B_ between *M. chiloensis* (13.111‰^2^, 95% CI [12.52, 13.70]) and *T. brasiliensis* (14.184‰^2^, 95% CI [13.00, 15.36]) (Figure 2). In January (early austral summer), we observed 34% overlap between the species isotopic niches, which increased to 50.4% in February. We also found that *T. brasiliensis* had a larger SEA_B_ than *M. chiloensis* in January (*M. chiloensis*: 3.184‰^2^, 95% CI [2.65, 3.72]; *T. brasiliensis*: 9.555‰^2^, 95 CI [8.16, 10.95], *p* = .01). The mean area of isotopic niche was similar for both species in February, although *M. chiloensis* was slightly more likely to have higher values than *T. brasiliensis* when comparing SEA_B_ (*M. chiloensis*: 17.87‰^2^, 95% CI [14.23, 21.51]; *T. brasiliensis*: 10.383‰^2^, 95% CI [9.24, 11.53], *p* = .087).

**FIGURE 3:**
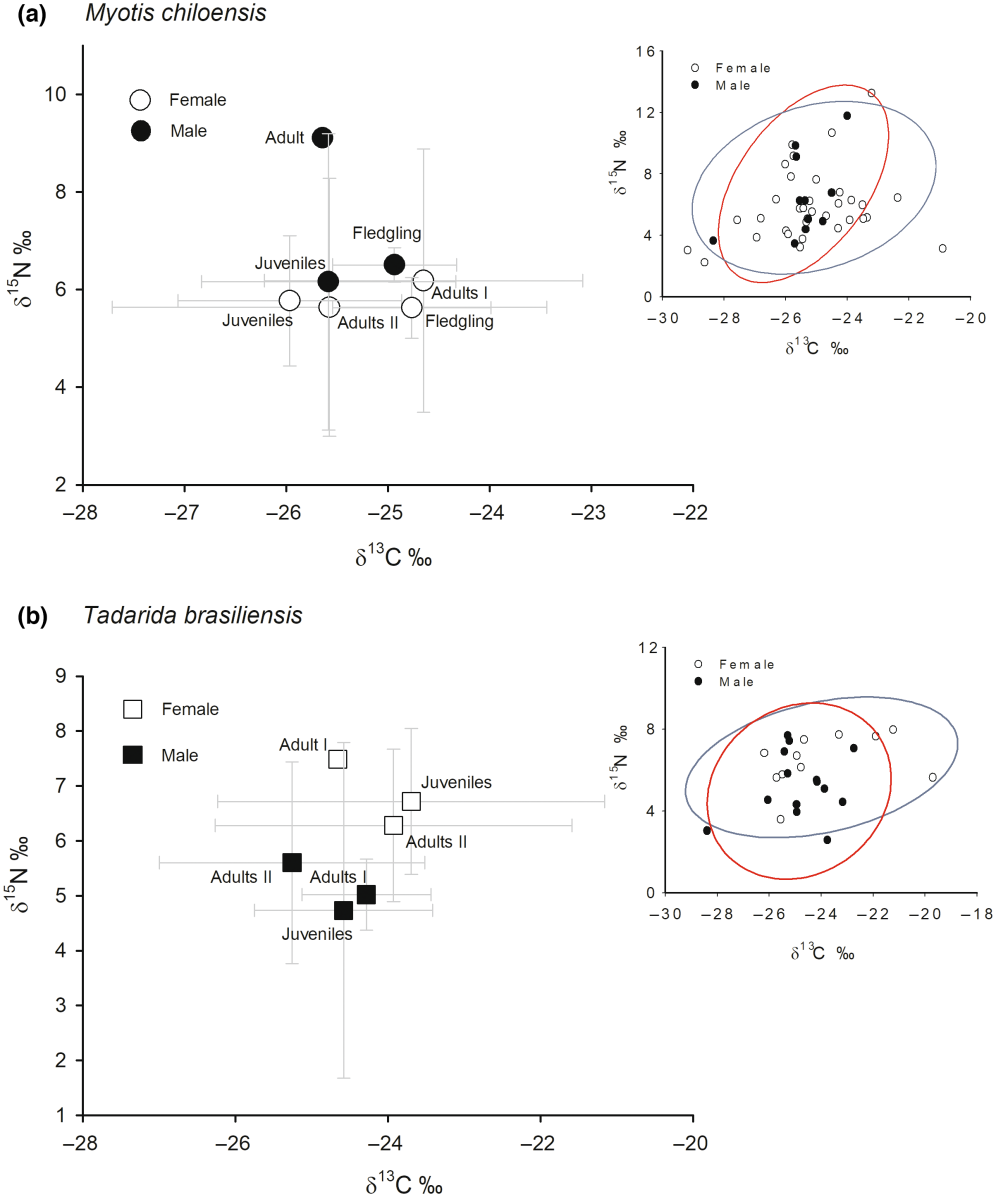
Stable isotope ratios of δ^15^N and δ^13^C (mean ± SD) from feces of two sympatric bat species in Chile separated by sex, age, and month (I = January, II = February). (a) *Myotis chiloensis* and (b) *Tadarida brasiliensis*. Inset plots (upper right corner) represent the isotopic niches (SEA_C_) separated by sex.

The comparison of isotopic niches between sexes revealed an overlap of 74% in *M. chiloensis*, with no significant differences in the size of SEA_B_ between females (11.51‰^2^, 95% CI [10.19, 12.83]) and males (13.82‰^2^, 95% CI [12.32, 15.32], *p* = .264). For this species, a high SEA_C_ overlap (98.2%) was observed between January and February, with a significantly larger SEA_B_ in the former month (January: 17.348‰^2^, 95% CI [13.86, 20.84]; February: 9.541‰^2^, 95% CI [8.99, 10.09], *p* = .04, Figure 3a).

In *T. brasiliensis*, the comparison of isotopic niches between sexes revealed an overlap of 64.6% with no significant differences in the size of SEA_B_ between the sexes (female: 7.381‰^2^, 95% CI [6.13, 8.73]; male: 9.016‰^2^, 95% CI [7.39, 10.61], *p* = .329). A high SEA_C_ overlap (98.8%) was observed between January and February with a larger SEA_B_ in the former (January: 10.442‰^2^, 95% CI [8.38, 12.50] February: 3.238‰^2^, 95% CI [2.48, 3.40], *p* = .01), as shown in Figure 3b.

**FIGURE 4:**
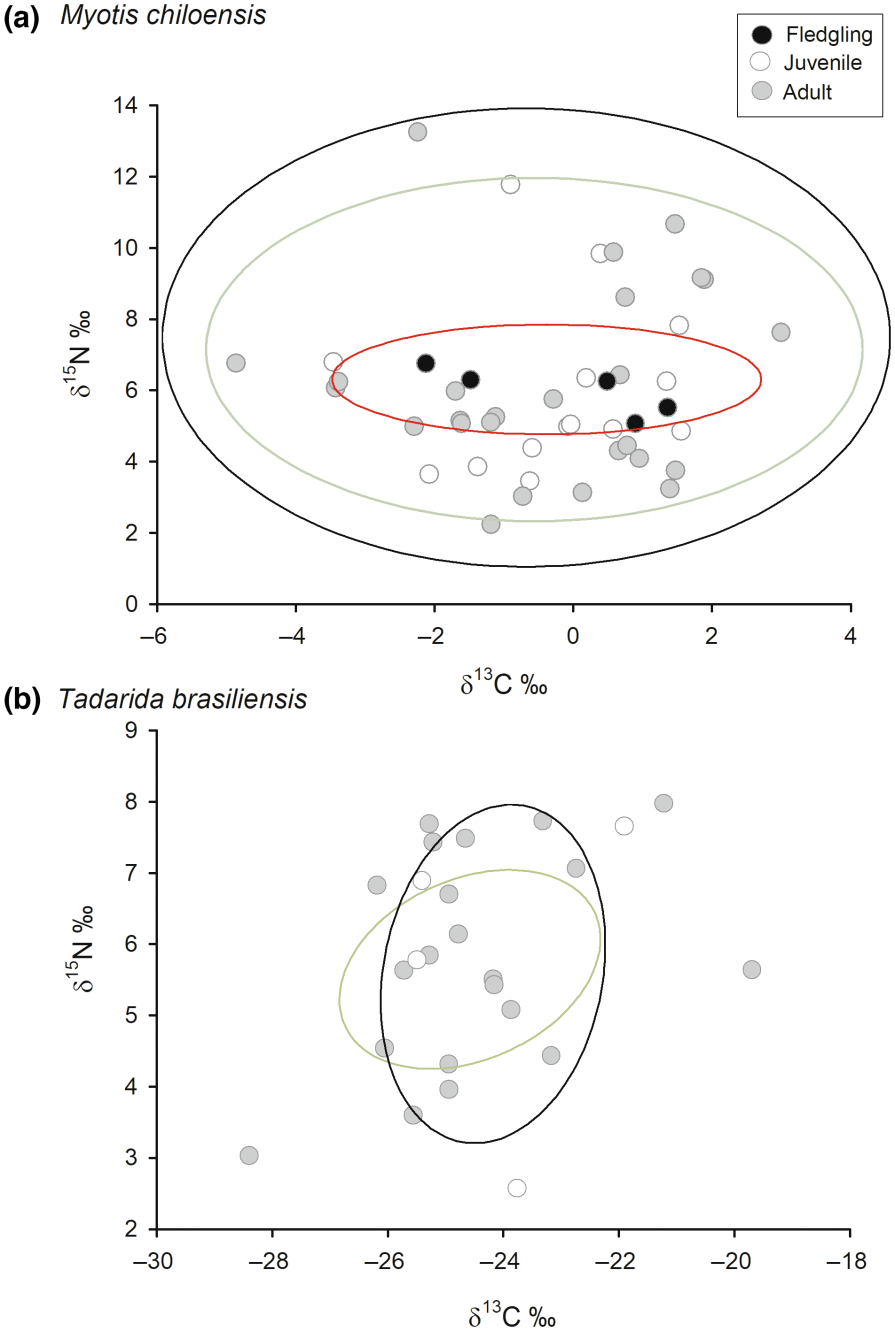
Isotopic niches (corrected standard ellipses; SEA_C_) determined from fecal samples of two sympatric bat species in Chile of different ages. (a) *Myotis chiloensis* and (b) *Tadarida brasiliensis*. Black for fledgling, open for juvenile, and gray for adult.

The age effect on isotopic signatures in *M. chiloensis* showed a nested aggregation of ellipses throughout ontogeny (Figure 4a). Specifically, SEA_C_ in fledglings (3.041‰^2^, 95% CI [1.55, 4.53]) accounted for 8.93% of the juvenile ellipse, with no significant differences in SEA_B_ size (*p* = .875). Juvenile SEA_B_ (11.530‰^2^, 95% CI [9.82, 13.24]) was nested within the adult SEA_B_ (16.527‰^2^, 95% CI [15.22, 17.83]) with 47.04% overlap, indicating a lower standard ellipse area (*p* = .001). In *T. brasiliensis*, we observed 22% of isotopic niche overlap, with significantly lower SEA_B_ in juveniles (juvenile SEA_B_: 7.445‰^2^, 95% CI [5.73, 9.16]; adult: 31.555‰^2^, 95% CI [23.22, 39.89], *p* = .003; see Figure 4b).

The age effect on isotopic signatures in *M. chiloensis* showed a nested aggregation of ellipses throughout ontogeny (Figure 4a). Specifically, SEA_C_ in fledglings (3.041‰^2^, 95% CI [1.55, 4.53]) accounted for 8.93% of the juvenile ellipse, with no significant differences in SEA_B_ size (*p* = .875). Juvenile SEA_B_ (11.530‰^2^, 95% CI [9.82, 13.24]) was nested within the adult SEA_B_ (16.527‰^2^, 95% CI [15.22, 17.83]) with 47.04% overlap, indicating a lower standard ellipse area (*p* = .001). In *T. brasiliensis*, we observed 22% of isotopic niche overlap, with significantly lower SEA_B_ in juveniles (juvenile SEA_B_: 7.445‰^2^, 95% CI [5.73, 9.16]; adult: 31.555‰^2^, 95% CI [23.22, 39.89], *p* = .003; see Figure 4b).

### 3.3 Effects of body mass, morphology, and sex on isotopic signatures

We found no significant differences in body mass between males and females in both species (*T. brasiliensis*: *F*_(1,23)_ = 0.403, *p* = .532; *M. chiloensis*; *F*_(1,41)_ = 0.353, *p* = .555). In *T. brasiliensis* we observed a positive correlation between higher δ^15^N values and heavier individuals among females (*r* = .554; permutation test: *p* = .032). In *T. brasiliensis*, the sex of the individual emerged as the strongest predictor of δ^15^N values in the GLMM analysis (*p* < .01).

**FIGURE 5:**
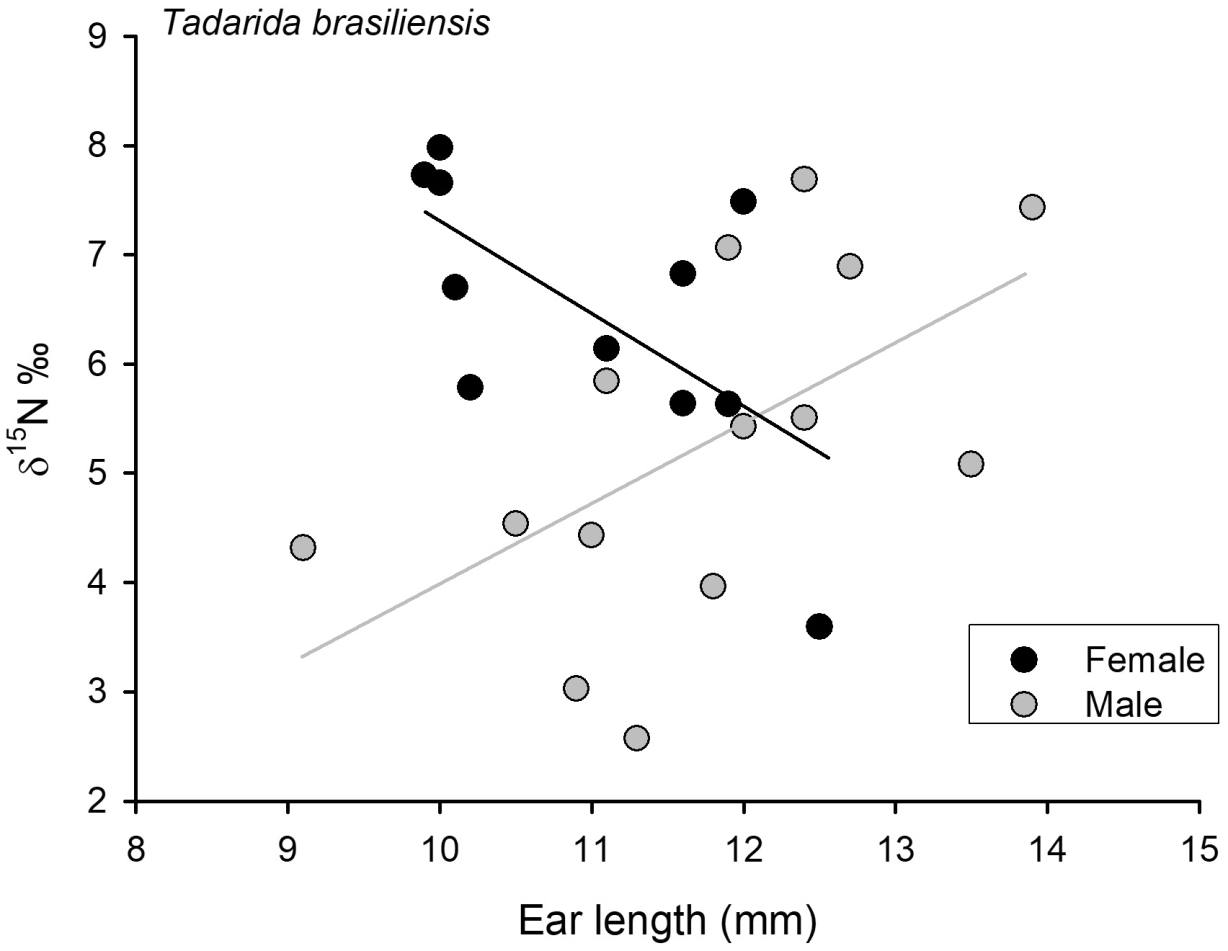
Association between ear length and δ^15^N values, for females and males of *Tadarida brasiliensis* in Chile.

When considering species as a random variable in GLMMs, ear length exhibited a weak effect on δ^15^N in pooled data (*p* = .046, see Table 2). A specific analysis indicates a sex effect in the associations between ear length and δ^15^N in *T. brasiliensis*. In this species, ear length and δ^15^N values were positive for males (*r* = .570; *p* = .033) but negative for females (*r* = −.644; *p* = .032, Figure 5). When using pooled data, we identified a strong effect of body mass and wing loading on δ^13^C values while considering species as a random variable in GLMM analysis (*p* = .003 and *p* = .009, respectively; see Table 2).

**TABLE 2:** Generalized linear mixed models (GLMM) were used to analyze the influence of intrinsic and extrinsic variables on δ^15^N and δ^13^C values from the feces of two sympatric bats in Chile, *Tadarida brasiliensis* and *Myotis chiloensis*.

Specie	Variable	Model	Random variable	Variables	Estimates	*p*	AICc	Delta AICc
*T. brasiliensis*	δ^15^N	Ear length * Sex	Age	Ear length	−0.857	.02	278.2	0
				Sex	−19.209	0		
				Ear length: Sex	1.588	0		
		Ear length * Sex + Wing loading	Age	Ear length	−0.877	.012	279.97	1.27
				Sex	−19.573	0		
				Wing loading	11.51	.094		
				Ear length: Sex	1.61	0		
	δ^13^C	Ear length * Sex + Moonlight intensity	Age	Ear length	−0.952	.07	113.29	5.27
				Sex	−15.386	.038		
				Moonlight intensity	2.793	.153		
				Ear length: Sex	1.269	.051		
		Body mass: Sex	Age	Body mass: Female	0.13	.046	109.91	1.89
				Body mass: Male	0.076	.166		
*M. chiloensis*	δ^15^N	Moonlight intensity: Sex	Age	Moonlight intensity: Female	3.757	.004	211.24	2
				Moonlight intensity: Male	−4.994	.008		
		Total length + Tragus length + Ear length + Moonlight intensity	Sex	Total length	0.139	.119	171.27	0
				Tragus length	0.782	.085		
				Ear length	−0.644	.046		
				Moonlight intensity	−5.214	.001		
	δ^13^C	Wing length	Date	Wing length	0.239	.064	153.35	2.72
		Body mass + Wing loading	Date	Body mass	1.093	.093	155.71	5.09
				Wing loading	−45.542	.06		
*T. brasiliensis & M. chiloensis*	δ^15^N	Total length + Tragus length + Ear length + Moonlight intensity	Specie	Total length	167.3	.119	171.27	0
				Tragus length	0.782	.085		
				Ear length	−0.644	.046		
				Moonlight intensity	−5.214	.001		
		Total length * Moonlight intensity		Total length	−0.051	.411	273.6	0
			Date	Moonlight intensity	−18.755	.009		
				Total length: Moonlight intensity	0.27	.029		
	δ^13^C	Total length + Body mass + Wing loading	Specie	Total length	−0.061	.194	253.37	1.83
				Body mass	1.082	.003		
				Wing loading	−44.342	.009		
		Body mass + Wing loading	Sex	Body mass	0.923	.008	252.59	1.061
				Wing loading	−41.503	.015		

### 3.4 Effect of moonlight

When pooled data were analyzed, a strong negative relationship was identified between moonlight intensity and δ^15^N values (*r* = −.346; *p* = .003), while no such relationship was observed for δ^13^C (*r* = −.043; *p* = .722). The potential impact of moonlight intensity was further supported when considering the sampling date as a random effect (*p* = .002, see Table 2). Subsequently, these associations exhibited differential representation between species. Specifically, *M. chiloensis* exhibited a negative association with both δ^15^N and δ^13^C (*r* = −.420; *p* = .004; *r* = −.326; *p* = .027, respectively). In contrast, *T. brasiliensis* showed weaker and nonsignificant correlations for both δ^15^N and δ^13^C (*r* = −.188, *p* = .369; *r* = .053; *p* = .801, respectively).

**FIGURE 6:**
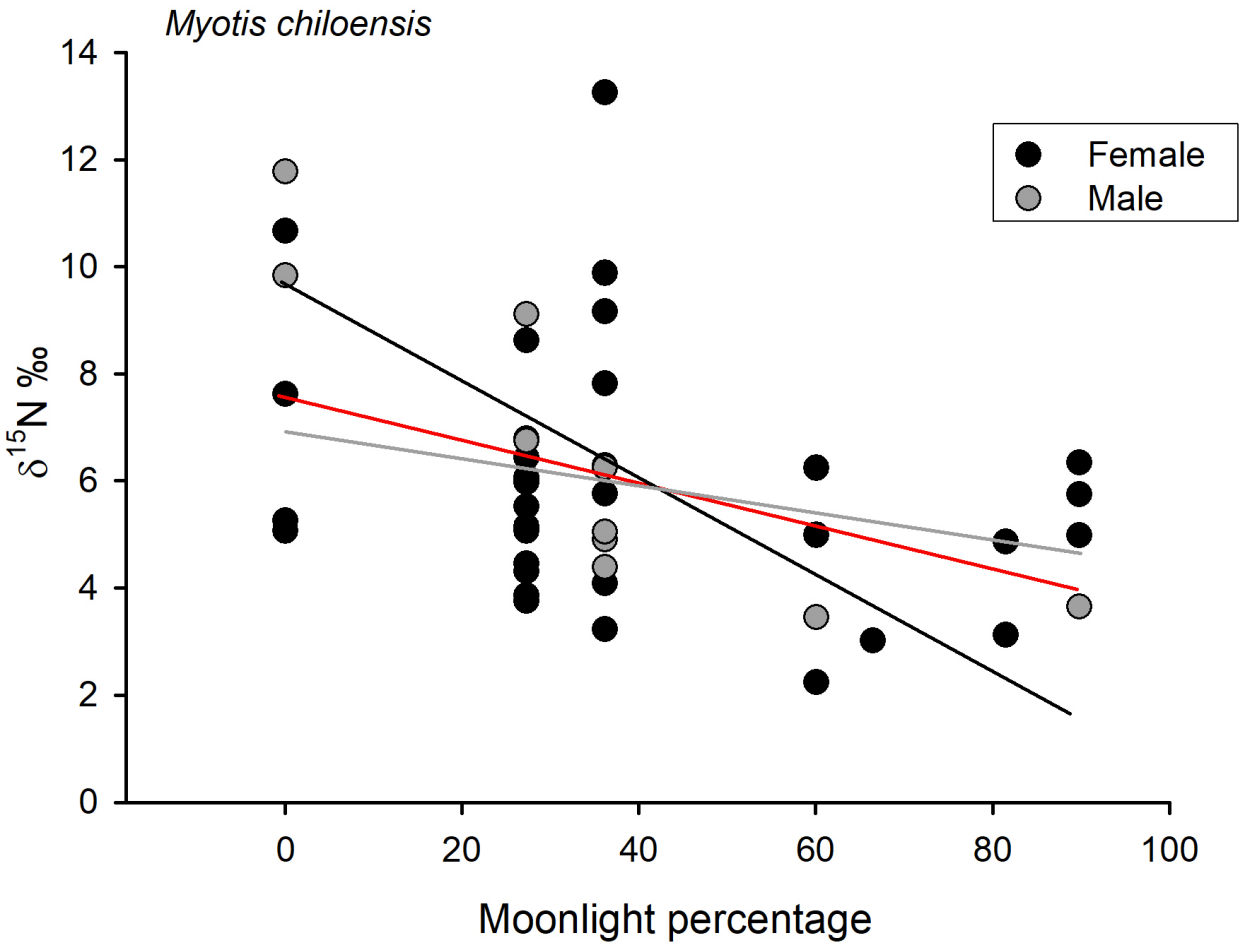
Correlation between moonlight percentage and δ^15^N values for *Myotis chiloensis* feces in Chile. Significant associations are detected only in pooled female–male data (red line) and in males alone (males: gray line; females: black line).

Further, the analysis by sex revealed that only males of *M. chiloensis* exhibited a negative and significant relationship between moonlight intensity at the time of capture and δ^15^N values (*r* = −.839; *p* = .001, Figure 6). Consistent with these findings, when considering the age of individuals as a random variable, a strong interaction between moonlight intensity and sex was identified (see Table 2).

## 4 DISCUSSION

Previous studies have shown that morphological features in combination with stable isotope analysis can provide insights into the structure of bat communities (Monadjem et al., 2018). In our study, we aimed to assess the effects of several factors that may influence the variability of resource use among insectivorous bats from a mixed colony, using isotopic signatures obtained from fecal samples. Our findings indicate that both intrinsic and extrinsic factors play important roles in shaping the isotopic niche overlap and resource use among bats. We found that morphology, age, sex, temporality, and moonlight are significant factors that influence bat foraging and prey selection.

### 4.1 Effect of intrinsic variables on isotopic niches

We found that body mass and relative ear length were the main morphological features associated with δ^15^N variation in fecal samples. Higher body mass apparently led to greater feasibility of acquiring prey at higher trophic levels in *T. brasiliensis*, as indicated by the higher values of δ^15^N.

Our data also suggest that ear length has different expressions depending on sex. *Tadarida brasiliensis* males, with larger ears, exhibit higher δ^15^N values, in contrast to females, who show an inverse relationship. This result could be linked to morphological or behavioral differences between sexes, which requires further exploration, and therefore should be interpreted with caution. Tragus and ear length also play vital roles in locating and capturing insect prey, influencing foraging behavior and acoustic structure (Fenton & Bogdanowicz, 2002; Giménez et al., 2023; Stannard et al., 2020). However, with some exceptions, large ears are typically associated with bats employing slow flight and a gleaning strategy (see López‐González & Ocampo‐Ramírez, 2021; Norberg & Rayner, 1987). Consequently, gleaning bats—which rely less on echolocation for hunting and instead depend on prey‐generated noise—are thought to have larger ears that aid in prey capture through passive listening. For example, *M. bechsteinii*, with significantly larger ears than *M. nattereri*, exhibits superior abilities in detecting and localizing low‐frequency rustling sounds from prey (Siemers & Swift, 2006). Indeed, it has been reported that in phyllostomid bats, notable differences occur in ear morphology between species in different functional diet categories, particularly those representing the challenges of capturing mobile and evasive prey versus nonmobile and nonevasive prey (Leiser‐Miller & Santana, 2020).

Nevertheless, as suggested by Bloch et al. (2011), body size alone may be insufficient to determine niche differentiation and species coexistence at least in New World bats. We identified sex as a factor that may explain isotopic variation and resource use, but temporally extended studies, or the use of different tissues that capture different temporal variations in isotopic incorporation, should be conducted to confirm the consistency of sex differences in resource use. It is worth noting that stable isotopes in fecal samples can reveal recent dietary inputs, typically within 2–3 h of food ingestion (Salvarina et al., 2013), although this duration can be longer (10–20 h) in certain cases (Schattanek et al., 2021).

To the best of our knowledge, this study is the first to examine the effect of ontogeny on isotopic signatures in bat feces, despite other reports identifying nitrogen isotope ratios (δ^15^N) in tissues from different ontogenetic stages (see Roswag et al., 2014). Our study revealed a nested overlap of isotopic signatures in *M. chiloensis*, indicating that older individuals have a broader isotopic niche and likely have a more diverse diet. These differences may be attributed to several factors, such as changes in bite force (Santana & Miller, 2016), expansion of foraging range, or modification of foraging time with older age (see Adams & Pedersen, 2000; and references therein). This finding is significant considering that similar studies have not considered age as a factor to explain the variation in the feeding habits of bats.

The extent to which differences in the width of the isotopic niche among age classes can be attributed to variations in diet‐to‐tissue isotopic composition (referred to as ∆diet‐tissue), resulting from different metabolic pathways during growth, remains unclear (Kadye et al., 2020). Notably, studies conducted on invertebrates, such as the shrimp *Neomysis integer*, have shown that inter‐individual variability in muscle isotopic signatures decreases as individuals grow, leading to a reduction in standard ellipse area values (Gorokhova, 2018). However, when it comes to ∆diet‐feces in birds and mammals, including bats, it can be considered negligible (Kuwae et al., 2022; Salvarina et al., 2013). Therefore, we anticipate a lesser impact of age classes, influenced by differences in growth rates, on isotopic metrics.

### 4.2 External variables on isotopic signal

Our study provides new evidence regarding the effect of moonlight intensity on bat foraging habits. Indeed, our results suggest that bats prey on higher trophic levels when moonlight intensity is lower. We identified a significant correlation between moonlight intensity and δ^15^N, which may be attributed to the influence of moonlight on bat spatial use and their ability to access to different insect types (Gomes et al., 2020; Roeleke et al., 2018).

Moonlight has been shown to restrict bats from feeding in shadows or to reducing activity altogether (Reith, 1982). Previous studies have reported that bat species generally adjust their activity based on moonlight intensity, although the effects are species‐specific. For instance, the activity of *M. chiloensis* is lower during bright nights, whereas *T. brasiliensis* is the only species whose activity is higher during bright nights (Vásquez et al., 2020). The reduced activity of *M. chiloensis* could attributed to hazard avoidance (e.g., the presence of raptors, see Mikula et al., 2016) or differential prey availability depending on light intensity. In another instance, Lang et al. (2006) found that white‐throated round‐eared bats (*Lophostoma silvicolum*) and bush crickets (Orthoptera) were significantly more active during dark periods associated with a new moon, compared to bright periods under full moon. Thus, it is likely that bat foraging activity is chiefly influenced by prey availability, and that the reasons for lunar phobia may differ among species (Gomes et al., 2020; Roeleke et al., 2018) as well as in response to the presence of artificial lighting at night (Cravens et al., 2018; Minnaar et al., 2015).

Nevertheless, our results about the effect of moonlight intensity on bat foraging need to be regarded with caution due to the limited sampling period and the potential autocorrelation between moonlight intensity and season. Besides, our sampling period considered only the austral summer and was performed during a limited range of moonlight‐intensity exposures. Then, further efforts are needed to determine the effect of light exposure while discriminating the seasonal effect and evaluating species‐specific responses.

### 4.3 Ecological implications

Our analysis indicates that both species of bats display variable degrees of niche overlap overtime during the austral summer. Moreover, we identified that isotopic signature variability was associated with differences in bat age, sex, morphology, and behavior. Previous studies analyzing isotopic signature overlap have highlighted mechanisms such as the movement capacity of bats, which may facilitate their coexistence (e.g., Ruadreo et al., 2018). Those reports are consistent with our observation that morphological traits of *M. chiloensis* and *T. brasiliensis*, such as wing load, could be associated with flight energy costs and other ecological characteristics (Canals et al., 2001). In a similar context, prior studies have emphasized spatial segregation as a factor that differentiates bat niches, facilitated by anatomical differences related to flight (e.g., wing morphology). This spatial separation enables them to coexist while foraging for different prey types (Magalhães de Oliveira et al., 2020; Moyo & Jacobs, 2020).

The observed differences between *M. chiloensis* and *T. brasiliensis* in their isotopic niche overlap and their behavior are noteworthy. Our study identified an increase in isotopic niche overlap in February compared to January, with part of the *T. brasiliensis* population migrating by the end of February, possibly due to a reduction of prey (Romano et al., 1999; Villa & Cockrum, 1962). Consequently, it is plausible that the remaining (non‐migrant) population overlaps more with *M. chiloensis* in their trophic (isotopic) niche because of the reduced prey availability at the end of summer. Additionally, food overlap increased between the sexes of *M. chiloensis* in February, also pointing to a reduction in prey supply. This explanation agrees with studies on fruit bats, which show a consistent pattern of reduced isotopic niche width during periods of low food availability (Shipley & Twining, 2020). Furthermore, they suggest that, under food reduction, mechanisms such as opportunistic foraging and spatiotemporal niche segregation may play a role in facilitating coexistence.

In closing, it seems important to continue addressing studies of trophic niches in mixed colonies of bats. Such studies should help identify which species may compete for food resources or show dietary responses to environmental changes (Ruadreo et al., 2018; Shipley & Twining, 2020), which could lead to changes in bat community structure. In addition, they may facilitate the design of effective conservation strategies for species with low trophic niche widths (Lam et al., 2013).

## AUTHOR CONTRIBUTIONS

**Isaac Peña‐Villalobos:** Conceptualization (equal); formal analysis (equal); investigation (equal); methodology (equal); writing – original draft (equal). **Catalina B. Muñoz‐Pacheco:** Conceptualization (equal); formal analysis (equal); investigation (equal); methodology (equal); writing – original draft (equal). **Martín A. H. Escobar:** Investigation (equal); methodology (equal); project administration (equal); resources (equal); supervision (equal); writing – review and editing (equal). **Fabian M. Jaksic:** Visualization (equal); writing – original draft (equal); writing – review and editing (equal). **Pablo Sabat:** Methodology (equal); project administration (equal); resources (equal); supervision (equal); writing – original draft (equal); writing – review and editing (equal).

## FUNDING INFORMATION

This work was funded by ANID FONDECYT 1200386; ANID PIA/BASAL FB0002; ANID—Millennium Science Initiative Program—Center Code NCN2021_050.

## Supporting information


Data S1

## Data Availability

Data that support the findings of this study are available as supporting material.
