# Supplementary material for: Living with voracious roommates: Factors that explain isotopic niche variation in a mixed colony of insectivorous bats
Source: Ecol Evol. 2024 Mar 17;14(3):e10939. doi: 10.1002/ece3.10939 (PMC10945080; doi:10.1002/ece3.10939)
Supplement: Supplementary file 1 — Data S1 [file ECE3-14-e10939-s001.zip › legends.docx]

"Stable isotope ratios (δ15N and δ13C) from feces and morphometric data of two sympatric bat species in Chile (*Myotis chiloensis*and*Tadarida brasiliensis*) separated by sex, age and date."
